# Supplementary material for: Multicenter Technical Validation of 30 Rapid Antigen Tests for the Detection of SARS-CoV-2 (VALIDATE)
Source: Microorganisms. 2021 Dec 15;9(12):2589. doi: 10.3390/microorganisms9122589 (PMC8704317; doi:10.3390/microorganisms9122589)
Supplement: Supplementary file 1 [file microorganisms-09-02589-s001.zip › microorganisms-1477829-supplementary.pdf]

Supplementary material

Supplementary tables

Supplementary Table S1. All antigen assays evaluated between 7 November 2020 and 23 February 2021 in alphabetic order according to the manufacturer's name.

Manufacturer	Name of antigen assay	Manufacturer's reference	Country of production	Certification	Target	Type of test
Acro Biotech	Acro COVID-19 Antigentest	INCP-ACO502	USA	CE	Nucleo-capsid and spike protein	Qualitative membrane-based immunoassay
AMEDA Labordiagnostic	AMP Rapid Test SARS-CoV-2 Ag	RT2952-S	Austria	CE	Nucleo-capsid protein	Immunochromatographic membrane assay with colloidal gold
Becton Dickinson	The BD Veritor™ System	256082	USA	CE-IVD	Nucleo-capsid protein	Immunochromatographic membrane assay
Beijing Hotgen Biotech Co., Ltd.	Novel Coronavirus 2019-nCoV Antigen Test (Colloidal Gold)	76749	China	CE	Not specified	Colloidal Gold Immunochromatographic assay
BioNote, Co., LTD.	NowCheck® COVID-19 Ag Test	RG1901DG (NP)	Korea	CE	Not specified	Rapid chromatographic immunoassay
BIOSYNEX Swiss	Biosynex COVID-19 Ag BSS^ (colloidal gold)	SW40006	Switzerland	CE-IVD	Nucleo-capsid protein	Colloidal gold immunochromatographic membrane assay
BIOSYNEX Swiss	Biosnyex COVID-19 Ag+ BSS° (agglutin)	N/A	Switzerland	N/A	N/A	N/A
Biozek Medical	COVID-19 Antigen Rapid Test Cassette	BCOV-502	Netherlands	CE-IVD	Not specified	Qualitative membrane-based immunoassay
GenBody Inc.	GenBody Covid-19 Ag	COVAG025	Korea	CE-IVD	Nucleo-capsid	Colloidal Gold

					protein	Immunochromatographic assay
Green Cross Medical Science Corp.	Genedia W COVID-19 Ag§	643G	Korea	CE-IVD	Not specified	Immunochromatographic membrane assay
Guangzhou Wondfo Biotech Co.	Wondfo SARS-CoV-2 Antigen Test	W196	China	CE	Not specified	Qualitative Lateral Flow Immunoassay
Hangzhou Clongene Biotech Co., Ltd.	CLUNGENE® COVID-19 Rapid Antigen Test Cassette	ICOV5002	China	CE	Nucleo-capsid protein	Lateral flow immunoassay
HANGZHOU LYSUN Biotechnology	LYSUN SARS-CoV-2 Antigen	COV-201	China	CE-IVD	Not specified	Double antibody sanwich method with colloidal gold
Hangzhou Realy Tech Co.	REALY antigen test	K511416D	China	CE	Not specified	Immunochromatographic membrane assay with colloidal gold
Healgen Scientific Limited Liability Company	CLINITEST®, Rapid COVID-19 Antigen Test+	GCCOV-502a	USA	CE-IVD	Nucleo-capsid protein	Immunochromatographic membrane assay
Humasis	Humasis COVID-19 Ag Test	N/A	Korea	CE-IVD	Nucleo-capsid and RBD protein	Immunochromatographic membrane assay with colloidal gold
Lansion Biotechnology Co.	Lansionbio® COVID-19 Antigen Test Kit (Dry Fluorescence Immunoassay)	N/A	China	CE-IVD	Nucleocapsid protein	Dry fluorescence immunoassay

Lepu Medical Zechnology Co., Ltd.	SARS-CoV-2 Antigen Rapid Test (Colloidal Gold Immunochromatography)	N/A	China	CE-IVD	Nucleo-capsid protein	Colloidal Gold Immunochromatographic assay
LumiraDx AB	LumiraDx SARS-CoV-2 Ag	SPEC-32312 R5 ART-00571 R11	Sweden	CE-IVD	Nucleo-capsid protein	Rapid microfluidic immunofluorescence assay
MaiMed GmbH	MEDsan® SARS-CoV-2 Antigen Rapid Test	76748	Germany	CE-IVD	Not specified	Solid-phase immunochromatography test
MP Biomedicals GmbH	MP COVID-19 Antigen Rapid Test	07AG6020B	Germany	CE-IVD	Not specified	Double antibody sandwich immunochromatographic lateral flow device
Newgene Hangzhou Bioengineering	COVID-19 Antigen Detection Kit	9901-NCOV-01G	China	CE-IVD	Nucleo-capsid protein	Qualitative membrane-based immunoassay
Precision Biosensor, Inc.	Exdia COVID-19 Ag	PR-FC13	Korea	CE	Nucleo-capsid protein	Time-resolved fluorescence immuno-chromatography assay
PRIMA Lab SA	COVID-19 Antigen Rapid Test	N/A	Switzerland	CE-IVD	Not specified	Immunochromatographic membrane assay
Shenzhen Microprofit Biotech Co.	Fluorecare® SARS-CoV-2 Spike Protein Test Kit	MF-60 and MF-63	China	CE-IVD	Spike protein	Colloidal Gold Chromatographic Immunoassay (MF-60) and Fluorescence Immunoassay (MF-63)
Sugentech Inc.	SGTi-flex COVID-19 Ag	N/A	Korea	CE	Not specified	Gold nanoparticle-based Immunochromatographic assay
VivaCheck Biotech (Hangzhou) Co., Ltd	VivaDiag™, SARS-CoV-2 Rapid Ag Test	VCD05-01-013	China	CE-IVD	Nucleo-capsid protein	Lateral flow immunoassay

Willi Fox	Willi Fox COVID-19 Antigen Test®	7771417	Switzerland	CE	Nucleo-capsid protein	Qualitative membrane-based immunoassay
Wuhan UNscience Biotechnology Co., Ltd.	SARS-CoV-2 Antigen Rapid Test Kit	N/A	China	CE-IVD	N/A	Colloidal gold Immunoassay
Xiamen Boson Biotech Co., Ltd.	Medicovid-AG® Test	16923451	Germany	CE-IVD	Nucleo-capsid protein	Colloidal Gold Chromatographic Immunoassay

CE: conformity to the European Directive 98/79/EC; IVD: conformity to the In Vitro Diactostics directive of the Federal Food, Drug, and Cosmetic Act; N/A: not available.

Supplementary Table S2. List of samples used for cross-reactivity validation. Each PCR was performed in a technical triplicate and the quantification was calculated from the mean value.

**Number**	**Material**	**Replicate 1 Ct-value**	**Replicate 2 Ct-value**	**Replicate 3 Ct-value**	**Copies/mL**	**Log_{10} Copies/mL**
RV-NEG-6	CoV 229	25.3	25.5	25.7	110200	5.0
RV-NEG-7	CoV 229	24.9	25.2	25.7	105700	5.0
RV-NEG-8	CoV 229	25.1	26.4	26.4	73700	4.9
RV-NEG-9	CoV HKU1	41.4	neg	neg	100	2.0
RV-NEG-10	CoV HKU1	29.9	30.1	30.8	6400	3.8
RV-NEG-11	CoV HKU1	39.3	39.6	41.1	100	2.0
RV-NEG-12	CoV OC43	21.7	21.9	22.1	2171000	6.3
RV-NEG-13	CoV OC43	25.3	25.7	25.7	187500	5.3
RV-NEG-14	CoV OC43	23.0	23.4	23.8	815400	5.9
RV-NEG-15	CoV NL63	21.5	21.8	22.0	4756100	6.7

RV-NEG-16	CoV NL63	24.6	24.9	26.1	472400	5.7
RV-NEG-17	CoV NL63	25.3	25.5	25.5	367800	5.6
RV-NEG-18	Parainfluenza 1	32.2	32.7	33.0	4900	3.7
RV-NEG-19	Parainfluenza 1	27.0	27.3	27.9	133900	5.1
RV-NEG-20	Parainfluenza 1	22.9	23.2	23.5	182300	5.3
RV-NEG-21	Parainfluenza 2	26.8	27.2	27.6	172400	5.2
RV-NEG-22	Parainfluenza 2	27.5	27.6	28.0	120400	5.1
RV-NEG-23	Parainfluenza 2	25.1	25.7	25.7	555900	5.7
RV-NEG-24	Parainfluenza 3	30.3	31.1	31.1	9700	4.0
RV-NEG-25	Parainfluenza 3	24.3	24.3	24.3	684100	5.8
RV-NEG-26	Parainfluenza 3	26.5	26.9	27.2	131100	5.1
RV-NEG-27	Parainfluenza 4	23.3	23.6	23.6	433200	5.6
RV-NEG-28	Parainfluenza 4	25.9	26.3	26.5	93300	5.0
RV-NEG-29	Parainfluenza 4	27.6	27.7	27.2	53900	4.7
RV-NEG-30	Rhino/Enterovirus	neg	neg	neg	neg	neg
RV-NEG-31	Rhino/Enterovirus	neg	neg	neg	neg	neg
RV-NEG-32	Rhino/Enterovirus	neg	neg	neg	neg	neg
RV-NEG-33	Rhino/Enterovirus	neg	neg	neg	neg	neg
RV-NEG-34	Rhino/Enterovirus	neg	neg	neg	neg	neg
RV-NEG-30	Rhino/Enterovirus	24.4	24.5	24.8	41800	4.6
RV-NEG-31	Rhino/Enterovirus	21.8	22.0	22.1	286300	5.5
RV-NEG-32	Rhino/Enterovirus	21.7	21.8	21.8	1492000	6.2
RV-NEG-33	Rhino/Enterovirus	32.1	32.5	32.6	1300	3.1

RV-NEG-34	Rhino/Enterovirus	27.4	27.7	27.7	86200	4.9
RV-NEG-35	Influenza A	24.0	24.1	24.2	136100	5.1
RV-NEG-36	Influenza A	23.1	23.3	23.3	385000	5.6
RV-NEG-37	Influenza A	24.4	24.5	24.7	117000	5.1
RV-NEG-38	Influenza A	25.5	25.6	26.0	62300	4.8
RV-NEG-39	Influenza A	24.6	24.6	24.7	119000	5.1
RV-NEG-40	Influenza A	25.2	25.3	25.5	50200	4.7
RV-NEG-41	Influenza B	22.5	22.6	22.7	200500	5.3
RV-NEG-42	Influenza B	23.3	23.5	23.5	78600	4.9
RV-NEG-43	Influenza B	22.7	22.8	29.2	136100	5.1
RV-NEG-44	Influenza B	24.6	24.8	24.9	30500	4.5
RV-NEG-45	Influenza B	24.0	24.0	24.1	50500	4.7
RV-NEG-46	Influenza B	23.7	24.2	25.3	33800	4.5
RV-NEG-47	RSV-A/B	20.1	20.2	20.4	3675800	6.6
RV-NEG-48	RSV-A/B	23.0	23.1	23.4	602500	5.8
RV-NEG-49	RSV-A/B	21.7	21.9	22.0	1464800	6.2
RV-NEG-50	RSV-A/B	24.9	25.0	25.1	139000	5.1
RV-NEG-51	RSV-A/B	21.8	22.2	22.2	891300	6.0
RV-NEG-52	RSV-A/B	24.9	24.9	25.4	104000	5.0
RV-NEG-53	Metapneumovirus	22.9	23.4	23.8	203900	5.3
RV-NEG-54	Metapneumovirus	22.4	22.6	22.9	339800	5.5
RV-NEG-55	Metapneumovirus	24.1	24.1	24.5	107700	5.0

Supplementary Table S3. Minimal threshold of detection for rapid antigen tests on serial dilution from positive samples of a cell culture super-natant and a positive clinical sample.

Manufacturer	Name of antigen assay	Cell culture super-natant	Positive clinical sample.
Acro Biotech	Acro COVID-19 Antigentest	Failed	Passed
AMEDA Labordiagnostic	AMP Rapid Test SARS-CoV-2 Ag	Passed	Passed
Becton Dickinson	The BD Veritor™ System	Passed	Passed
Beijing Hotgen Biotech Co., Ltd.	Novel Coronavirus 2019-nCoV Antigen Test (Colloidal Gold)	Failed	Failed
BioNote, Co., LTD.	NowCheck® COVID-19 Ag Test	Passed	Passed
BIOSYNEX Swiss	Biosynex COVID-19 Ag BSS^ (colloidal gold)	Passed	Passed
BIOSYNEX Swiss	Biosnyex COVID-19 Ag+ BSS° (agglutin)	Passed	Passed
Biozek Medical	COVID-19 Antigen Rapid Test	Passed	Failed

	Cassette		
GenBody Inc.	GenBody Covid-19 Ag	Failed	Passed
Green Cross Medical Science Corp.	Genedia W COVID-19 Ag§	Failed	Failed
Guangzhou Wondfo Biotech Co.	Wondfo SARS-CoV-2 Antigen Test	Failed	Passed
Hangzhou Clongene Biotech Co., Ltd.	CLUNGENE® COVID-19 Rapid Antigen Test Cassette	Passed	Passed
HANGZHOU LYSUN Biotechnology	LYSUN SARS-CoV-2 Antigen	Passed	Passed
Hangzhou Realy Tech Co.	REALY antigen test	Passed	Passed
Healgen Scientific Limited Liability Company	CLINITEST®, Rapid COVID-19 Antigen Test+	Passed	Passed
Humasis	Humasis COVID-19 Ag Test	Passed	Passed
Lansion Biotechnology Co.	Lansionbio® COVID-19 Antigen Test Kit (Dry Fluorescence Immunoassay)	Passed	Passed
Lepu Medical Zechnology Co., Ltd.	SARS-CoV-2 Antigen Rapid Test (Colloidal Gold	Failed	Failed

	Immunochromatography)		
LumiraDx AB	LumiraDx SARS-CoV-2 Ag	Passed	Passed
MaiMed GmbH	MEDsan® SARS-CoV-2 Antigen Rapid Test	Passed	Passed
MP Biomedicals GmbH	MP COVID-19 Antigen Rapid Test	Passed	Passed
Newgene Hangzhou Bioengineering	COVID-19 Antigen Detection Kit	Passed	Passed
Precision Biosensor, Inc.	Exdia COVID-19 Ag	Passed	Passed
PRIMA Lab SA	COVID-19 Antigen Rapid Test	Failed	Passed
Shenzhen Microprofit Biotech Co.	Fluorecare® SARS-CoV-2 Spike Protein Test Kit	Passed	Passed
Sugentech Inc.	SGTi-flex COVID-19 Ag	Passed	Passed
VivaCheck Biotech (Hangzhou) Co., Ltd	VivaDiag™, SARS-CoV-2 Rapid Ag Test	Passed	Passed
Willi Fox	Willi Fox COVID-19 Antigen Test®	Passed	Passed
Wuhan UNscience Biotechnology Co., Ltd.	SARS-CoV-2 Antigen Rapid Test Kit	Passed	Failed

Xiamen Boson Biotech Co., Ltd.	Medicovid-AG® Test	Passed	Passed

Supplementary figures.

Supplementary Figure S1. Distribution of Ct values overall (A) and across all four laboratories (B).

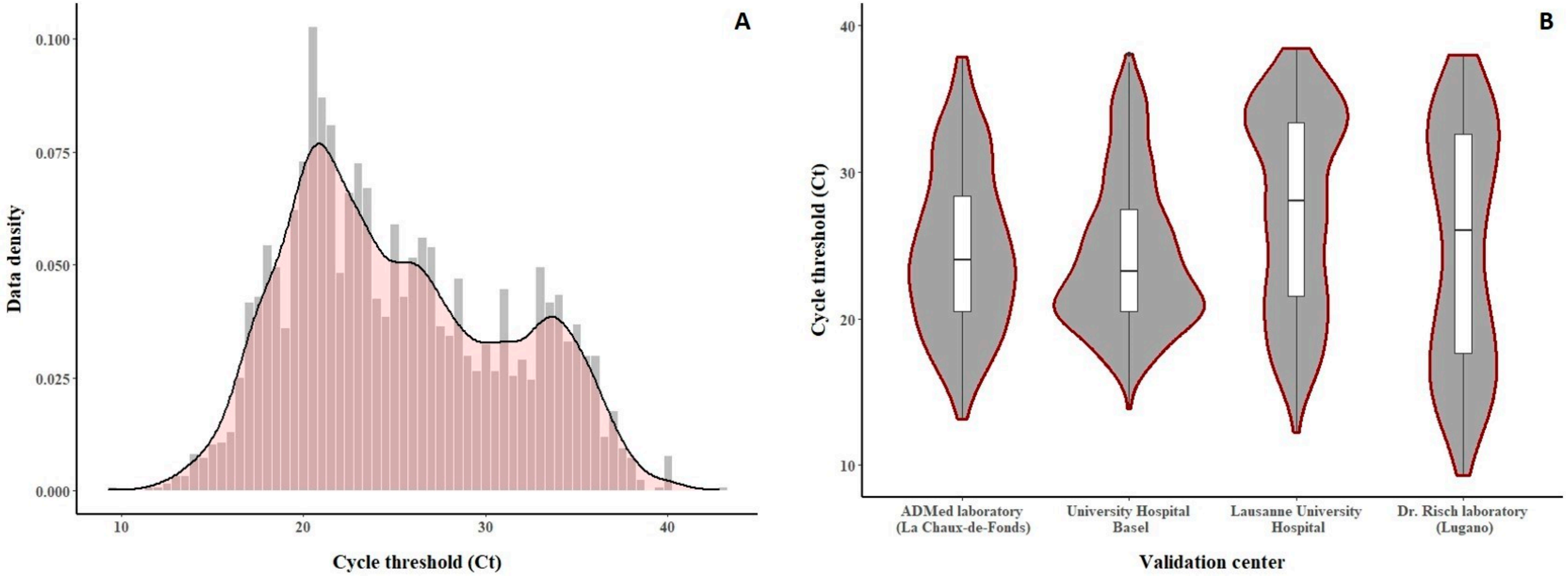

Figure legend

Supplementary Figure S1. Histogram of cumulated Ct values obtained overall in the study. B. Violins represent the Ct distribution of values in each validation center; horizontal black lines represent the median Ct for each validation center.
